# Excellent response to trametinib and low dose chemotherapy in a patient with rare RAF1 mutated sarcoma in a tertiary care center in India

**DOI:** 10.3332/ecancer.2025.1907

**Published:** 2025-05-15

**Authors:** Anjali Govind, Sameer Rastogi, Sunil Pasricha, Shamim Ahmed, Aastha Goel

**Affiliations:** 1 Department of Onco-Anesthesia and Palliative Medicine, All India Institute of Medical Sciences, New Delhi 110029, India; 2 Department of Medical Oncology, All India Institute of Medical Sciences, New Delhi 110029, India; 3 Department of Histopathology, Rajiv Gandhi Cancer Institute and Research Center, Delhi 110085, India; 4 Department of Nuclear Medicine, All India Institute of Medical Sciences, New Delhi 110029, India

**Keywords:** RAF1, sarcoma, trametinib, doxorubicin, S100, CD34

## Abstract

With the advent of next-generation sequencing, increasingly we can sub classify the soft tissue sarcomas into various subtypes with distinct prognostic and therapeutic implications. Sarcomas with RAF1 mutations are extremely rare and so far, the treatment strategies are not known. Here, we report a case of a 48-year-old lady who initially presented with right ear swelling, which was excised and was suggestive of dermatofibrosarcoma protuberans with S100 expression. After a disease-free interval of 25 months, the patient relapsed with metastasis in the lung. Repeat biopsy and next-generation sequencing (NGS) were suggestive of PDZRN3/RAF1 fusion mutated sarcoma. On presentation, the patient had an Eastern Cooperative Oncology Group performance status of 4 and had respiratory distress due to lung metastasis. After consensus and decision in the molecular tumour board, the patient was started on low-dose trametinib and doxorubicin. After three cycles of treatment, the patient had a partial response and post six cycles she had a near-complete response. This case exemplifies the value of molecular characterisation of soft tissue sarcoma and adds to the already sparse literature for RAF1 mutated sarcomas.

## Background

Sarcomas are a group of tumours that are derived from bone, muscle, cartilage and other connective tissues. The heterogeneity of these tumours is exhibited in their high cellular, molecular and genetic or epigenetic variability. Hence, the identification of single therapeutic targets can pose as a great challenge. With the recent wealth of molecular information, in some instances, revolutionary new driver mutations or oncogenic targets have been identified which help in the more effective targeted therapy with less toxicity [1].

Lucchesi *et al* [2] did a study in 2018 to characterising the targetable genomic alterations in soft tissue sarcomas in adult patients. In this study, 584 patients were included which consisted of 294 males and 290 females and the median age of the recruited patients for analysis was 56 years (range of 18 to 89 years). 57% of the patients had complex genomics sarcoma, 25% had translocation-related sarcomas while the rest 18% had other sarcomas with inactivating mutation. The most commonly altered genes in order of their frequency were TP53, MDM2, CDK4, RB1, ATRX, CDKN2A, PTEN, NF1, CDKN2B, KMT2D, GLI1, ATM, TERT, PI3KCA, NOTCH1, MAP2K4, ERBB4, ARID1A, TSC2 and TNFAIP3. The study concluded that 41% (239 cases) of all soft tissue sarcomas harboured at least 1 genomic alteration which was relevant clinically with a statistically significant higher number in other and complex genomics sarcomas than in the translocation related sarcomas. These had a potential toward personalised therapy [2].

More than 40 years ago, rapidly accelerated fibrosarcoma (RAF) was discovered as a cellular oncogene and since then 3 of its isoforms have been extensively studied. The RAF-MEK-ERK signal transduction pathway is a rat sarcoma (RAS) activated protein kinase cascade that has a role in the regulation of cell growth, proliferation and differentiation in response to certain growth factors, cytokines and hormones [3]. This pathway gets activated in malignancies, and they RAF kinases are considered as a potential therapeutic agent for targeted therapy [4].

RAF1 rearrangements have been commonly reported in pancreatic acinar cell carcinomas and studies show the incidence to be ranging from 14.3% to 18.5% [5]. The Cancer Genome Atlas PanCancer Atlas samples observed that there were 2.3% RAF1 alterations [6]. Hence, these alterations are being extensively studied for precise oncology due to their contribution to mitogen activated protein kinase (MAPK) pathway dysregulation [7,8].

We present a case report of a patient with metastatic RAF1 gene mutation sarcoma who was successfully treated with a combination of trametinib and doxorubicin.

## Case presentation

**Figure 1.**
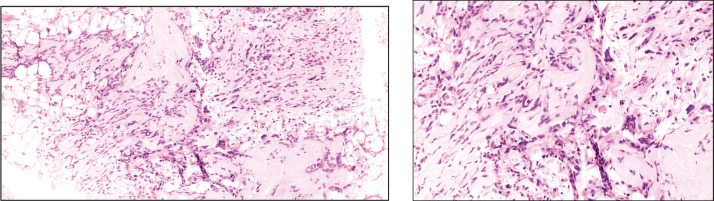
(a): Tru-cut biopsy shows proliferation of neoplastic spindle cells admixed with collagenous tissue and infiltrating adipose tissue. (b): Neoplastic spindle cells exhibiting low-grade atypia with inconspicuous mitosis and occasional multinucleated cells (arrow).

**Figure 2.**
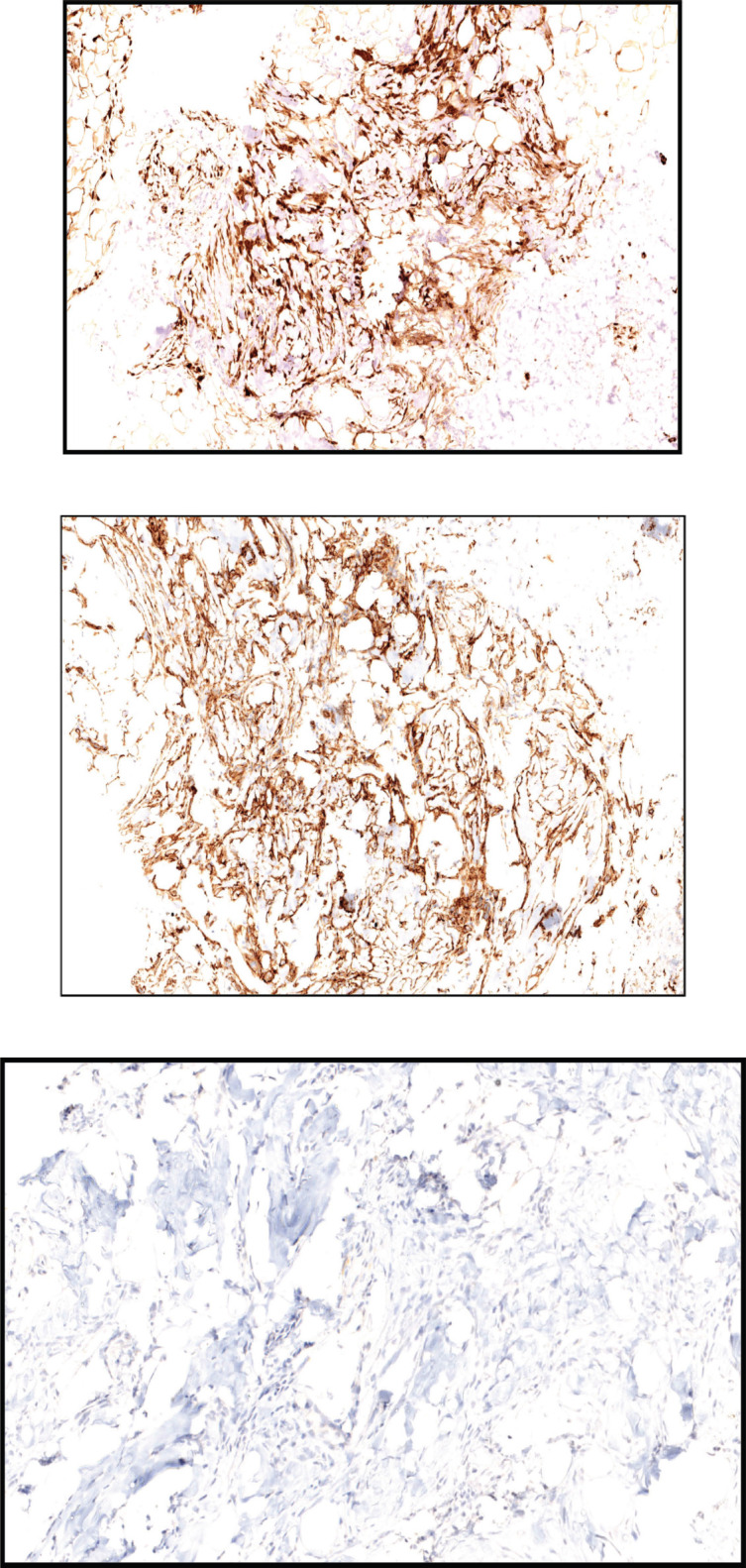
(a): Tumour cells show diffuse positivity for S100. (b): Tumour cells show diffuse positivity for CD34. (c): Tumour cells are negative for PAN-TRK.

A 48-year-old female patient presented with complaints of swelling behind the right ear. Biopsy was done which showed fascicles of spindle cells with mild atypia and sparse mitoses with interspersed hyalinisation suggestive of spindle cell neoplasm (FNCLCC grade 1) (Figure 1a and b) with immunohistochemistry (IHC) S100 and CD 34 positive and Negative for SMA, STAT6, PAN-TRK and SOX10 (Figure 2–c)

Wide local excision of the tumour was done in August 2021. Post-operative specimen showed sections of deep dermal and subcutaneous nodules with monotonous spindle cells arranged in short storiform patterns with intersecting fascicles at places, a few Touton giant cells present around the periphery of the nodules. Mitosis variable uptil 8–10/hpf. The tumour cells were positive for Vimentin, CD34 and S-100 positive, while negative for SOX 10, EMA, SMA and Melan A. Ki 67 was approximately 11%. All surgical margins were free.

The patient subsequently received adjuvant External Beam Radiation Therapy over 3 months and was free of disease during the next 2 years after which she developed a fever associated with generalised body pain. A chest X ray was done which showed the presence of a few rounded nodules. To confirm this, she underwent computed tomography (CT) of the chest which showed a left lower lobe mass lesion measuring 7.2 × 9.1 × 6.5 centimetres (cm), other nodules in the right upper lobe, right lower lobe, left lower lobe and few mediastinal lymph nodes.

**Figure 3.**
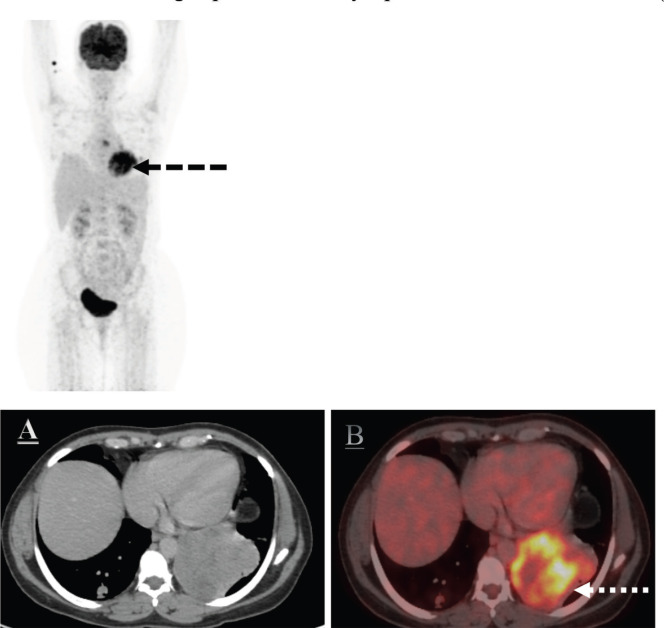
The axial CT and fused PET/CT images (a, b white dotted arrow and c, d white dotted arrow) represent the metabolic activity of the left lung lower lobe mass and nodule in the right lung lower lobe on the baseline PET/CT scan.

Positron emission tomography-computed tomography (PET-CT) scan was done for further evaluation. Metabolically active heterogeneously enhancing large soft tissue mass in the left lung lower lobe measuring 7.8 × 8.5 cm was noted. The mass was abutting mediastinal costal diaphragmatic pleura with adjacent left pleural thickening and mild left pleural effusion. Fluorodeocyglucose avid left hilar subcarinal lymph node measuring 1.0 × 1.9 cm subcentimetric right paratracheal lymph nodes were also noted (Figure 3).

Ultrasound-guided biopsy was done from the lung mass. Histopathology report showed spindle cells with moderate nuclear pleomorphism arranged in interlacing bundles with fascicular and focal herringbone patterns. Mitosis was 6–8/hpf. Necrosis seen in 20% tissue. On IHC, tumour cells were positive for Vimentin, CD34 and S-100, while negative for STAT6, SMA and CK. Ki -67 was 20%–25%.

The patient underwent molecular testing using a laboratory-developed, CAP-accredited NGS-based Soft Tissue Sarcoma Panel from MedGenome Labs, which accurately identifies single nucleotide variants (SNVs), small insertions and deletions (InDels), and gene fusions in 101 genes curated using World Health Organisation 2021 and the latest National Comprehensive Cancer Network (NCCN) guidelines. Fusions are screened using RNA sequencing, and therefore, all known and unknown partners are detected with 98% sensitivity. A histopathology review was carried out on the FFPE blocks selected for profiling to determine the tumour content and tumour genomic RNA was extracted from formalin-fixed paraffin-embedded (FFPE) tissue block and used to perform targeted gene capture using a custom hybrid capture kit. Reportable alterations and fusions were prioritised, classified and reported based on the Association for Molecular Pathology–American Society of Clinical Oncology–College of American Pathologists guidelines and NCCN guidelines. The limit of detection for SNVs is 5% and indels is 10% variant allele frequency, for fusions is >10 spanning reads.

**Figure 4.**
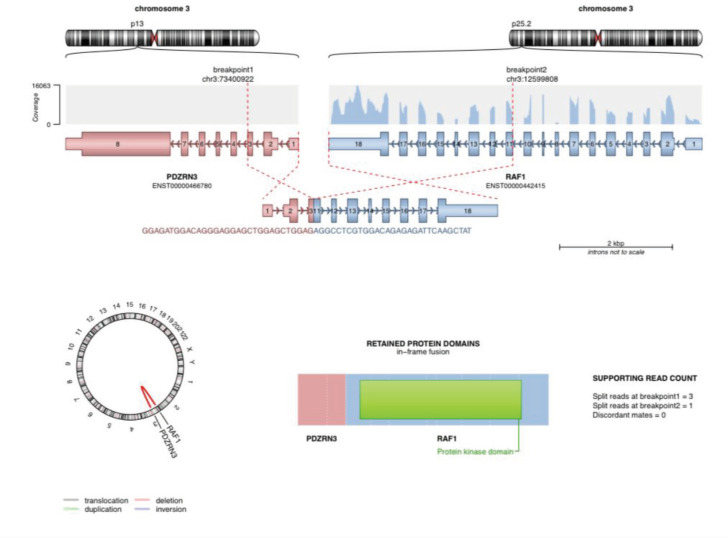
The image shows PDZRN3/RAF1 fusion detected using NGS-based soft tissue sarcoma panel from MedGenome Labs.

The mutation of potential clinical significance noted in our patient was: *PDZRN3/RAF1* fusion (as depicted in Figure 4).

During the disease trajectory patient developed complaints of shortness of breath (Modified Medical Research Council grade 4) and heaviness in the left chest for which inpatient management was done. The patient had eastern cooperative oncology group performance status of 4. She was initiated on oxygen therapy. Chest X ray was done which was suggestive of gross left-sided pleural effusion for which thoracocentesis was done and haemorrhagic fluid was drained. Subsequently, intercostal drainage tube was inserted in the left pleural cavity due to rapidly accumulating large-volume effusion. Eventually, pleurodesis of the left pleural cavity was done. She was initiated on BIPAP support in view of increased respiratory effort.

**Figure 5.**
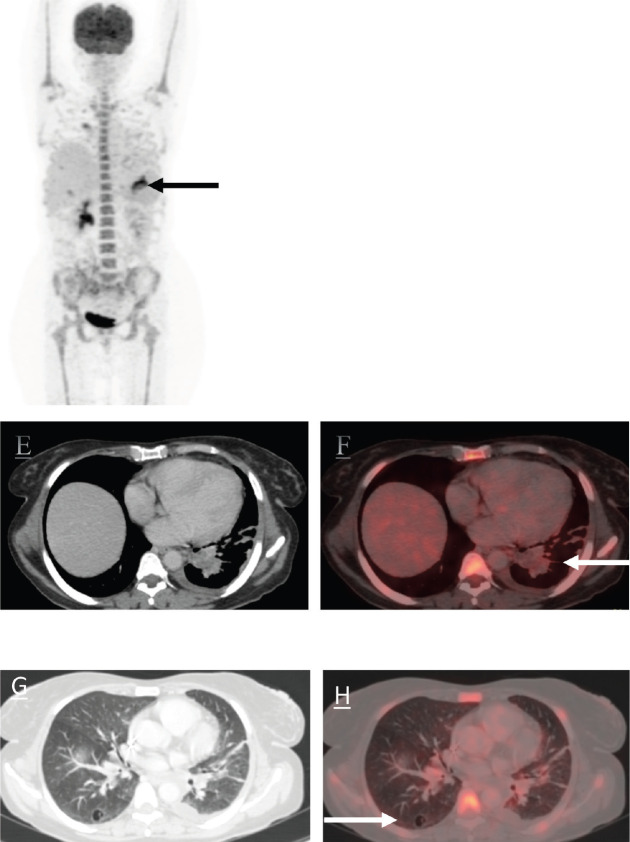
The MIP images (I, black arrow) and (II, black dotted arrow) represent the mass in left lung lower lobe. There is an interval reduction in the size of the mass. The axial CT and fused PET/CT images (e, f white arrow and g, h white arrow) represent significant reduction in size and metabolic activity of the left lung lower lobe mass and nodule in right lung lower lobe which now shows cavitation.

The patient was started on Tab trametinib 1.5 milligram (mg) OD and Inj doxorubicin (60 mg/m^2^) 6 cycles 3 weekly. Patient showed an excellent response to the above regimen with interval reduction in the size and metabolic activity of the mass (as depicted in Figure 5). Patient had grade 1 anemia and paronychia after the initiation of these regimens. There were no other drug toxicities or adverse effects which were noted. She had symptomatic improvement and improved performance status and oxygen therapy was gradually tapered off. She was ultimately discharged. The patient is on regular follow up of 3 months till date and is doing well.

## Discussion

RAF1 belongs to the RAF family of signalling kinases, which are downstream of RAS and ultimately activate the MEK–ERK pathway. This pathway causes cell proliferation and survival of the cancer cells. A study by Suurmeijer *et al* [9] evaluated spindle cell tumours with CD34 and S 100 coexpression in the absence of SOX 10 and the tissue was subjected to RNA sequencing. 25 patients were evaluated and the gene fusion and rearrangements which were seen were PDZRN3-RAF1, SLAMP-RAF1, TMF1-RAF1, RAF1, BRAF, LMNA-NTRK1, TPM3-NTRK1, TPR-NTRK1 and SPECC1L-NTRK2 [9].

Out of the 25 patients, 8 patients had RAF1 gene fusion or rearrangements. Two cases were present in children (2 and 10 years of age) and 6 occurred in adult patients with ages ranging from 27 to 67 years. The location of the tumours varied from abdomen, shoulder, back, chest wall, thigh and rectum.

On histopathological examination, all these tumours showed S100 and CD 34 co-expression which ranged from patchy or multifocal to diffuse and strong. All the tumours were consistently negative for SOX 10. Microscopically most of the tumours showed infiltrative growth patterns within subcutaneous fat, skeletal muscle and viscera. The tumours also showed bland spindle morphology with patternless architecture and scant mitotic activity. A pattern of stromal collagen deposition which included keloidal bands and perivascular rings was also noted.

Out of the eight patients, 37.5% of them showed increased cellularity while the rest of them showed presence of low cellularity. On follow up of these eight patients with RAF1 positive sarcomas, four patients showed no evidence of disease while one patient with PDZRN3–RAF1 gene rearrangement on follow up of 7 months was found to be alive with disease with lung, liver and peritoneal metastasis. The follow up data of the remaining three patients was not available [9].

In another case report of a paediatric spindle cell neoplasm in a 4-year-old boy with tumour in the thigh with S100 and CD 34 co-expression has been described showing PDZRN3-RAF1 gene fusion in whom excision of the tumour was done. The excised specimen had negative margins. Further imaging showed no evidence of disease or distant metastasis. The child was tumour disease free on 8 months follow up post-surgery and there was no adjuvant treatment given [10].

Another case report has been published which revealed a novel MTAP-RAF1 fusion in a 51-year-old male patient which included the tyrosine kinase domain of RAF1 in a patient with soft tissue sarcoma (not otherwise specified) of foot. Wide local excision of the tumour was done in this case and there was no adjuvant treatment given [11]. A summary table (Table 1) has been incorporated to concisely present the findings from previous case reports.

Trametinib is a reversible, highly selective allosteric inhibitor of MEK1/MEK2 (also known as MAP2K1 and MAP2K2) activation and kinase activity. In enzymatic and cellular studies, Trametinib has shown to inhibit the kinase activity of MEK1 and MEK2, prevented RAF-dependent MEK phosphorylation and prolonged inhibition of phosphorylated ERK (a substrate of MEK). This leads to blocking of cell proliferation and inducing apoptosis [12].

Trametinib should, in theory, be effective in treating sarcomas with RAF1 mutations, nevertheless, there is exceedingly rare data regarding the same. A case report of a patient with advanced chemotherapy-refractory myxofibrosarcoma harboring RAF1 S259P mutation and CDKN2A/B loss showed complete radiological response on treatment with MEK inhibitor Trametinib and CDK4/6 inhibitor Palbocilib. This combination was well tolerated by the patient and showed clinical improvement as well. Tumour clearance was also confirmed by the circulating tumour DNA monitoring [13].

Studies have shown that Trametinib is fairly well tolerated and the most common adverse events witnessed are rash and diarrhoea which are easily managed. Although on dose escalation beyond the maximum tolerable daily dose (more than 3 mg/day) can cause central serous retinopathy. Less than 1% of patients also had treatment-related grade 3 left ventricular dysfunction or a decrease in ejection fraction [14]. None of the anticipated side effects were encountered in our patient.

The greatest potential for the use of Trametinib is possibly in combination with both targeted and cytotoxic chemotherapies in tumours that contain genetic alterations in the MAPK activating pathways. There is research undergoing to check the efficacy of incorporating the inhibition of the MAPK pathway along with BRAF inhibitors [15].

To conclude, the presented case highlighted the significance of molecular testing and the effectiveness of targeted therapy in spindle cell sarcoma. The functional significance and consequences of this genetic fusion remain elusive due to the paucity of literature and need to be further studied, which can have an impact over the refinement of diagnosis, prognostic indicators and targeted therapy.

## Conflicts of interest

The authors declare they have no conflicts of interest.

## Funding

No funding was received for this study.

## Figures and Tables

**Table 1.** Summary of RAF1 sarcomas from review of literature.

Case	Age/Sex	Location	Gene fusion/ rearrangement	Follow up
1	67/F	Abdomen	PDZRN3-RAF1	Alive with disease (7 months)- lung, liver and peritoneal metastasis [9]
2	41/M	Thigh	SLMAP-RAF1	Not available [9]
3	2/F	Rectum	TMF1-RAF1	Not available [9]
4	45/F	Back	RAF1	No evidence of disease (36 months) [9]
5	10/M	Thigh	RAF1	Not available [9]
6	38/F	Shoulder	RAF1	No evidence of disease (9 months) [9]
7	27/F	Back	RAF1	No evidence of disease (26 months) [9]
8	59/M	Chest wall	RAF1	No evidence of disease (25 months), s/p RT [9]
9	4/M	Thigh	PDZRN3-RAF1	No evidence of disease (8 months), s/p surgical excision [10]
10	51/M	Foot	MTAP-RAF1	No evidence of disease, s/p excision [11]
11	60/F	Myxofibrosarcoma breast with extensive liver metastasis	RAF1 S259P	No evidence of disease (8 months), post trametinib and palbocilib [13]
